# Using 16s rRNA sequencing to characterize the microbiome of tropical cutaneous ulcer disease: insights into the microbial landscape and implications for diagnosis and treatment

**DOI:** 10.1099/mgen.0.001234

**Published:** 2024-05-13

**Authors:** Becca L. Handley, Oliver Sokana, Kennedy Kwasi Addo, Josef Wagner, María Fookes, Emma Harding-Esch, Michael Marks, Nicholas R. Thomson, Ronan M. Doyle

**Affiliations:** 1 Faculty of Infectious Diseases, London School of Hygiene and Tropical Medicine, London, UK; 2 Solomon Islands Ministry of Health, Honiara, Solomon Islands; 3 Noguchi Memorial Institute for Medical Research, University of Ghana, Accra, Ghana; 4 Wellcome Trust Sanger Institute, Cambridge, UK; 5 Hospital for Tropical Diseases, London, UK; 6 University College London, London, UK

**Keywords:** 16S rRNA sequencing, cutaneous ulcer disease, *Haemophilus ducreyi*, yaws

## Abstract

Cutaneous ulcers are common in yaws-endemic areas. Although often attributed to '*Treponema pallidum* subsp. *pertenue'* and *Haemophilus ducreyi*, quantitative PCR has highlighted a significant proportion of these ulcers are negative for both pathogens and are considered idiopathic. This is a retrospective analysis utilising existing 16S rRNA sequencing data from two independent yaws studies that took place in Ghana and the Solomon Islands. We characterized bacterial diversity in 38 samples to identify potential causative agents for idiopathic cutaneous ulcers. We identified a diverse bacterial profile, including *Arcanobacterium haemolyticum*, *Campylobacter concisus*, *Corynebacterium diphtheriae*, *Staphylococcus* spp*.* and *Streptococcus pyogenes*, consistent with findings from previous cutaneous ulcer microbiome studies. No single bacterial species was universally present across all samples. The most prevalent bacterium, *Campylobacter ureolyticus*, appeared in 42% of samples, suggesting a multifactorial aetiology for cutaneous ulcers in yaws-endemic areas. This study emphasizes the need for a nuanced understanding of potential causative agents. The findings prompt further exploration into the intricate microbial interactions contributing to idiopathic yaw-like ulcers, guiding future research toward comprehensive diagnostic and therapeutic strategies.

**Impact Statement**

This study on the microbiome of cutaneous ulcer disease (CUD) significantly advances our understanding of the diverse microbial composition associated with CUD lesions in Ghana and the Solomon Islands. To our knowledge, to date there have only been two published studies investigating the causes of idiopathic cutaneous ulcers, both using samples collected in Papua New Guinea. Here, we have used a 16S ribosomal subunit sequencing to determine which bacterial species are present in each sample. The identification of potential causative agents such as *Staphylococcus* spp*.*, *Streptococcus* spp*.*, *Streptococcus pyogenes*, *Arcanobacterium haemolyticum* and *Corynebacterium diphtheriae* provides crucial insights for accurate diagnosis and targeted antimicrobial therapy. This study impacts clinical practice, as it underscores the need for refined tools to prevent misdiagnosis. Effective treatment will prevent the emergence antimicrobial resistance by limiting the use of inappropriate antibiotics.

## Data Summary

The 16S rRNA sequencing data used in this study are available at the European Nucleotide Archive under study accession number PRJEB11547; the accession numbers for the individual samples used in this study are provided in the Table S1, available with the online version of this article. The code used to analyse this data is described within Methods.

## Introduction

Tropical cutaneous ulcer disease (CUD) is an umbrella term to describe the presentation of exudative lesions common in the tropics [1]. Globally, yaws, caused by *Treponema pallidum* subsp. *pertenue* was until recently believed to be the major cause of CUD. However, the use of molecular diagnostics demonstrates up to 50 % of CUD cases are caused by *Haemophilus ducreyi* [2,3], with only 20–30 % caused by *T. pallidum* subsp*. pertenue*. One third of CUD lesions are not caused by either of these two agents and are considered idiopathic [2,4]. To date, there has been limited research on the causes of these idiopathic ulcers. Two studies have investigated potential aetiological agents; both focused on samples collected from individuals in Papua New Guinea [5,6]. The first study used metagenomic sequencing and the second used bacterial 16S rRNA sequencing techniques (hereafter 16S). Neither study found a dominant alternative CUD cause; however, the 16S microbiome study concluded that *Streptococcus pyogenes* was likely to have a causal role in CUD as it was the most abundant organism detected and appeared to be enriched in idiopathic ulcers. As yet, there have been no reports published investigating the potential causes of CUD in other yaws-endemic countries. An improved understanding of the microbiome of cutaneous ulcers in other settings could help identify causes of CUD and ultimately improve targeting of CUD antimicrobial therapy.

## Methods

This study used samples previously collected in 2014 and 2013 from studies in Ghana [7] and the Solomon Islands [2,8], respectively. In both studies, key demographic data, such as age and gender, were recorded, as well as clinical information about the lesion, such as lesion location and duration. Alongside this, rapid plasma reagin (RPA) assays, *T. pallidum* haemagglutination assays (TPHAs) and quantitative PCRs (qPCRs) for *T. pallidum* subsp*. pertenue* and *H. ducreyi* were performed. The limit of detection for both pathogens is 10–100 copies per reaction [9]. Results of initial testing for *T. pallidum* subsp*. pertenue* and *H. ducreyi* have been reported previously [2,7].

In this study, we used residual DNA to sequence the 16S rRNA gene. The V1–V2 region was amplified using the Illumina adapter and indexed PCR primers (forward, 5'-AGMGTTYGATYMTGGCTCAG-3', and reverse r356, 5'-GCTGCCTCCCGTAGGAGT-3'; from Eurofin Genomics). PCRs were performed in triplicate according to Illumina’s 16S metagenomic sequencing library preparation protocol [10] and sequenced using the 600 cycle MiSeq reagent kit V3 (Illumina). Sequences were uploaded to the European Nucleotide Archive under study accession number PRJEB11547, accession numbers for specific sequences in this study are shown in Table S1.

FastQ files were processed using a custom pipeline developed from a 16S benchmarking study [11]. In brief, flash2 [12] was used to join paired-end sequence reads. Sequences were then dereplicated, chimeric sequences were removed and the sequences were clustered at 98 % sequence similarity into operational taxonomic units (OTUs). Taxonomy was assigned using a multistep method. First, MAPseq [13] was used to compare OTUs to a reference V1–V2 16S rRNA gene from *H. ducreyi* (GenBank accession no. CP022037.2) and '*T. pallidum* subsp*. pertenue'* (GenBank accession no. CP104185.1). They were considered true matches if they showed at least 97 % nucleotide identity. Following this, all OTUs were compared 
to the full silva arb database [14] using sintax [15] and verified with results from MAPseq. The resulting taxonomic classifications were analysed with R using the Phyloseq package [16].

Reads that were not assigned to order level or those that were classed as eukaryotes or viruses were removed. We conglomerated reads that had the same species name and deleted OTUs comprised of fewer than 200 sequence reads. The top 15 OTU assignments were validated by aligning the amplicon sequence against the GenBank database using blast, available at National Center for Biotechnology Information [17]. We used the highest similarity GenBank search result as the correct assignment if it differed from that of the original analysis, provided the percentage of similarity was over 97 %.

## Results

Of the 38 participants included in the study, 16 were from Ghana and 22 were from the Solomon Islands. All participants were under 15 years old and the median age was 8 years. Of the 38 participants, 20 (52.6 %) were male (Table 1).

**Table 1.** Demographic data of the 38 participants included in the analysis

**Characteristic**	Overall	Ghana	Solomon Islands
No. of participants	38	16	22
Median age (years)	8	8	8
Age range (years)	5–14	6–12	5–14
Male sex (%)	20 (52.6)	11 (68.8)	9 (40.9)
Median lesion duration (weeks)	4.1	4.1	4.1
**Lesion location**			
Lower leg	20 (52.6)	0	20 (90.9)
Foot	2 (5.3)	0	2 (9.1)
Torso	10 (26.3)	10 (62.5)	0
Face	5 (13.2)	5 (31.3)	0
Arm	1 (2.6)	1 (6.25)	0

Conglomerating and filtering removed 298 980 reads (10.8 % of total reads) representing 37 219 OTUs (99.7 % of total OTUs). The final dataset contained 2 480 980 reads containing 94 OTUs representing 69 unique genera. All samples (*n*=38, 16 from Ghana and 22 from the Solomon Islands) had over 20 000 reads [median number of reads was 64, 72 (interquartile range: 49 371–86 943]. Overall, 78.6 % of the retained reads were represented by the 15 most prevalent OTUs. Three of the original most prevalent OTUs were *Janthinobacterium agaricidamnosum*, *Burkholderia contaminans* and *Curvibacter delicatus*. These were considered bacterial DNAs that are commonly found contaminating reagents found in DNA extraction kits [18]. They were removed from the downstream analysis [18].

In the original studies [2,7], none of the 38 samples had tested positive for the *H. ducreyi* hhDA qPCR target; however, in the current study there were 4 samples containing over 200 reads for the OTU assigned as *H. ducreyi*. One sample had detectable levels of *T. pallidum* subsp*. pertenue*, which correlates with the qPCR results of the previous study [7].

**Fig. 1.**
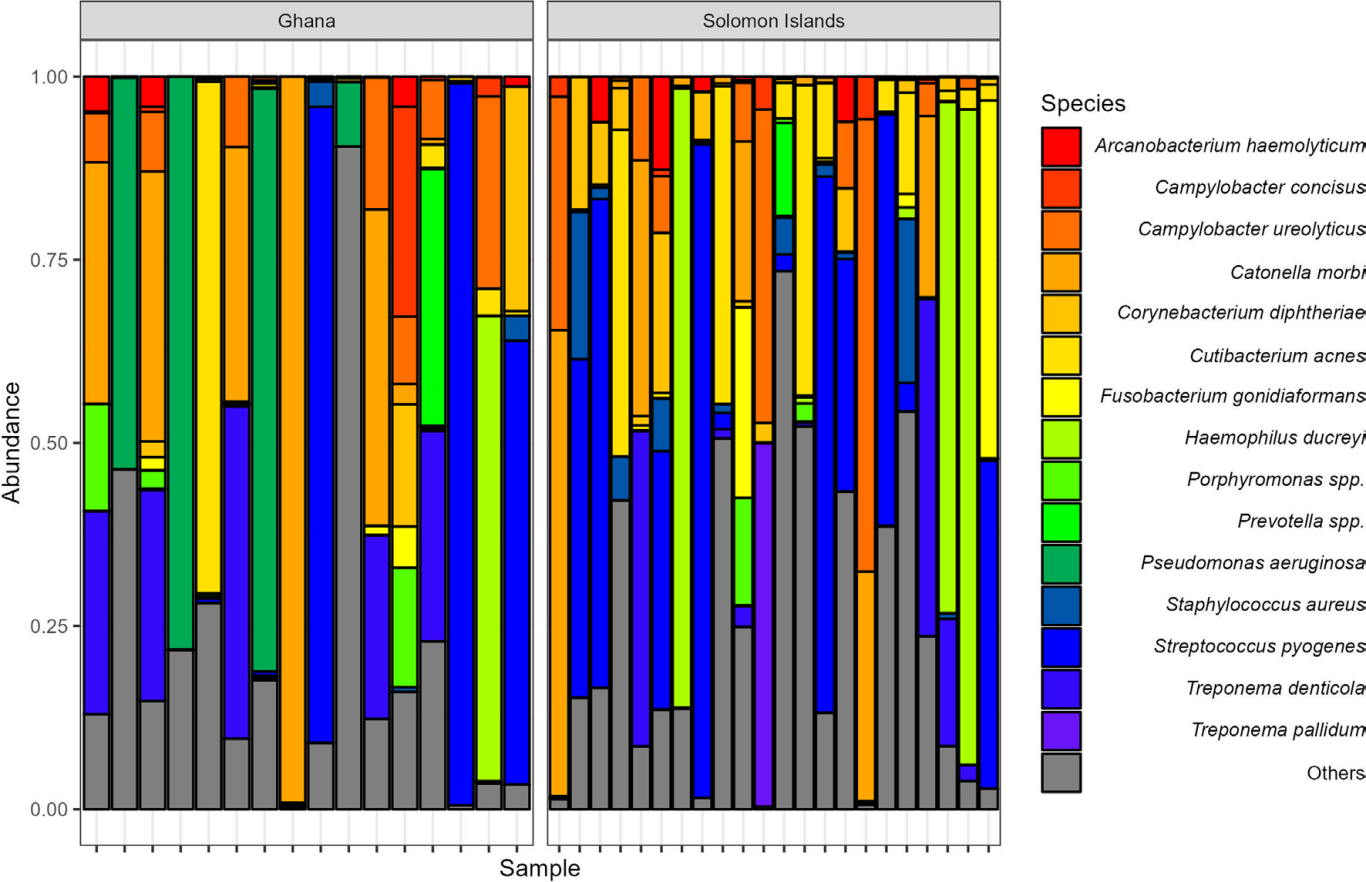
Relative abundance of the 15 most commonly assigned species/genera in 38 samples from Ghana (*n*=16) and the Solomon Islands (*n*=22).

**Fig. 2.**
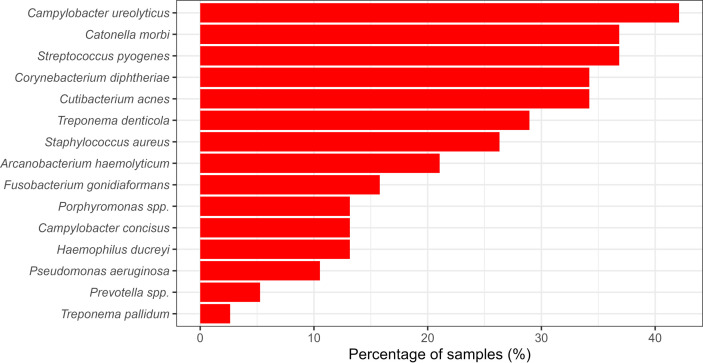
The proportion of samples from Ghana and the Solomon Islands (*n*=38) containing the 15 most prevalent OTUs. Samples were considered to contain the OTU if they accounted for at least 1 % of the total reads of the sample.

The other taxa identified in our study included common microbiota of the skin and dental cavity (Fig. 1). Alongside *T. pallidum* subsp*. pertenue* and *H. ducreyi*, some of the most common bacteria included *Arcanobacterium haemolyticum*, *Campylobacter concisus*, *Corynebacterium diphtheriae*, *Staphylococcus* spp*.*, *Streptococcus pyogenes* and *Treponema denticola*, all of which have been seen in previous cutaneous ulcer microbiome studies [5,6]. There was no common, shared microbiome across all samples collected. None of the 15 most prevalent OTUs were found in all samples tested (Fig. 2). The most prevalent bacterium, *Campylobacter ureolyticus*, was found in 42 % of samples.

## Discussion

This study identified a diverse range of bacteria in CUD samples collected in the Solomon Islands and Ghana. Four samples had detectable levels of *H. ducreyi*, contradicting the qPCR *H. ducreyi*-negative results of the previous study [7]. This discrepancy may be attributed to a mutation in the hhDA target primer binding site [19], resulting in false-negative qPCR results. Repeating these PCRs with an alternative *H. ducreyi* target could confirm this; however. this was not possible during this study as we did not have access to the residual samples.

Bacteria such as *Staphylococcus* spp. and *Streptococcus* spp., which were common in our analysis, could be causing skin conditions that may be confused with yaws or *H. ducreyi*. This highlights the importance of using molecular and serological tools to avoid misdiagnosis. We also detected a high prevalence of *Streptococcus pyogenes*, *A. haemolyticum* and *Corynebacterium diphtheriae*, which were found in 36, 34 and 21 % of the samples, respectively. These bacteria were proposed candidates as causes of chronic ulcers in Papua New Guinea [5,6] and should be further investigated as potential causes, or colonizers, of cutaneous ulcers.

This study was limited by the fact samples were collected at one time point without corresponding control swabs, either from asymptomatic skin of the individual with CUD, or from matched individuals without lesions. By investigating the microbiome of asymptomatic skin from the same individuals or their close contacts, it may be possible to differentiate between commensal bacteria and those that are potential causes of CUD. However, as this was a retrospective study using residual DNA, it was not possible to make this differentiation. Due to this being a retrospective observational study, adequate negative-control samples were not included in the sequencing run. Therefore, we are unable to determine which reads may have been extraction kit contaminants and which bacteria were truly present within the ulcer samples. However, by removing OTUs belonging to common kit contaminants [18] and OTUs with less than 200 reads, we hope to have filtered out most contaminating bacteria. Finally, in the Ghana study [7], researchers reported many participants had treated their ulcers with local topical treatments such as gentian violet paint, animal faeces or the contents of antibiotic capsules, which may have affected the bacterial composition of the sample. However, as samples were collected from children presenting with exudative lesions, we believe our 16S analysis gives a good indication of which bacteria were present at the time of sampling. Without a longitudinal study design, it is not possible to know which pathogens are responsible for causing the ulcers and which are merely opportunistic colonizers.

Future work should include microbiome analyses on larger sample sizes, participants from cohort studies and the inclusion of asymptomatic skin controls. This could include the use of novel technologies like the MinION sequencer (Oxford Nanopore Technologies) to allow for rapid, close-to-the-patient, or even point-of-care diagnosis of CUD. By inclusion of the appropriate controls, more inferences could be made about causes of cutaneous ulcers leading to the development of more appropriate diagnostic tests or strategies. Accurate diagnostic tools would enable the deliverance of appropriate antimicrobial treatments and help reduce the selection pressure for resistance development [20].

## supplementary material

10.1099/mgen.0.001234 Uncited Supplementary Material 1.

## Abbreviations

CUD: cutaneous ulcer disease
OTU: operational taxonomic unit
qPCR: quantitative PCR

## References

[1] González-Beiras C, Ubals M, Corbacho-Monné M, Vall-Mayans M, Mitjà O (2021). Yaws, *Haemophilus ducreyi*, and other bacterial causes of cutaneous ulcer disease in the South Pacific Islands. Dermatol Clin.

[2] Marks M, Chi K-H, Vahi V, Pillay A, Sokana O (2014). *Haemophilus ducreyi* associated with skin ulcers among children, Solomon Islands. Emerg Infect Dis.

[3] Mitjà O, Lukehart SA, Pokowas G, Moses P, Kapa A (2014). *Haemophilus ducreyi* as a cause of skin ulcers in children from a yaws-endemic area of Papua New Guinea: a prospective cohort study. Lancet Glob Health.

[4] González-Beiras C, Kapa A, Vall-Mayans M, Paru R, Gavilán S (2017). Single-dose azithromycin for the treatment of *Haemophilus ducreyi* skin ulcers in Papua New Guinea. Clin Infect Dis.

[5] Noguera-Julian M, González-Beiras C, Parera M, Ubals M, Kapa A (2019). Etiological characterization of the cutaneous ulcer syndrome in Papua New Guinea using shotgun metagenomics. Clin Infect Dis.

[6] Griesenauer B, González-Beiras C, Fortney KR, Lin H, Gao X (2021). *Streptococcus pyogenes* is associated with idiopathic cutaneous ulcers in children on a yaws-endemic island. mBio.

[7] Ghinai R, El-Duah P, Chi K-H, Pillay A, Solomon AW (2015). A cross-sectional study of “yaws” in districts of Ghana which have previously undertaken azithromycin mass drug administration for trachoma control. PLoS Negl Trop Dis.

[8] Marks M, Vahi V, Sokana O, Puiahi E, Pavluck A (2015). Mapping the epidemiology of yaws in the Solomon Islands: a cluster randomized survey. Am J Trop Med Hyg.

[9] Chen CY, Ballard RC (2012). The molecular diagnosis of sexually transmitted genital ulcer disease. Methods Mol Biol.

[10] Illumina (2013). 16S Metagenomic Sequencing Library Preparation. https://support.illumina.com/documents/documentation/chemistry_documentation/16s/16s-metagenomic-library-prep-guide-15044223-b.pdf.

[11] O’Sullivan DM, Doyle RM, Temisak S, Redshaw N, Whale AS (2021). An inter-laboratory study to investigate the impact of the bioinformatics component on microbiome analysis using mock communities. Sci Rep.

[12] Magoč T, Salzberg SL (2011). FLASH: fast length adjustment of short reads to improve genome assemblies. Bioinformatics.

[13] Matias Rodrigues JF, Schmidt TSB, Tackmann J, von Mering C (2017). MAPseq: highly efficient k-mer search with confidence estimates, for rRNA sequence analysis. Bioinformatics.

[14] Quast C, Pruesse E, Yilmaz P, Gerken J, Schweer T (2013). The SILVA ribosomal RNA gene database project: improved data processing and web-based tools. Nucleic Acids Res.

[15] Edgar RC (2016). SINTAX: a simple non-Bayesian taxonomy classifier for 16S and ITS sequences. bioRxiv.

[16] McMurdie PJ, Holmes S (2013). phyloseq: an R package for reproducible interactive analysis and graphics of microbiome census data. PLoS One.

[17] (2023). BLAST: Basic Local Alignment Search Tool. https://blast.ncbi.nlm.nih.gov/Blast.cgi.

[18] Salter SJ, Cox MJ, Turek EM, Calus ST, Cookson WO (2014). Reagent and laboratory contamination can critically impact sequence-based microbiome analyses. BMC Biol.

[19] Marks M, Fookes M, Wagner J, Ghinai R, Sokana O (2018). Direct whole-genome sequencing of cutaneous strains of *Haemophilus ducreyi*. Emerg Infect Dis.

[20] Hay SI, Rao PC, Dolecek C, Day NPJ, Stergachis A (2018). Measuring and mapping the global burden of antimicrobial resistance. BMC Med.

